# Xiaochaihu Decoction attenuates the vicious circle between the oxidative stress and the ALP inactivation through LPS-catecholamines interactions in gut, liver and brain during CCI_4_+ethanol-induced mouse HCC

**DOI:** 10.1186/1472-6882-13-375

**Published:** 2013-12-28

**Authors:** Xiao-qiu Liu, Xiao-jian Hu, Hong-Xing Xu, Xiao-Ying Zeng

**Affiliations:** 1 Piwei Research Institutes, Guangzhou University of Chinese Medicine, Guangzhou 510405, Guangdong, China; 2 The Fourth People’s Hospital, Zhanjiang 524008, Guangdong, China

**Keywords:** Xiaochaihu Decoction (XCHD), Ethanol, CCI_4_, Hepatocellular carcinoma (HCC), Liver depression and spleen deficiency (LDSD), Oxidative stress, Nitrosative stress, Lipopolysaccharid, Catecholamines, Alkaline phosphatase (ALP)

## Abstract

**Background:**

Xiaochaihu Decoction (XCHD) prevents hepatocarcinogenesis in association with inhibition of oxidative stress. However, alkaline phosphatase (ALP) activity, lipopolysaccharides (LPS)-catecholamines (CA) interactions in gut, liver and brain may play an important role in the status of oxidative stress. This study was to assess whether XCHD attenuates the vicious circle between oxidative stress and ALP inactivation through LPS-CA interactions.

**Methods:**

Hepatocellular carcinoma group (HCC) were induced by CCI_4_ + ethanol; HCC with Liver Depression and Spleen Deficiency (HCC + LDSD) were induced by squeezing tails (30 min/day), solitary breeding and intermittent fasting on the basis of HCC; XCHD was administered after 4 weeks of the HCC + LDSD. The degree of tissue injury were studied using a scoring system, and brain weights were measured. Peroxynitrite (ONOO^−^), malondialdehyde (MDA), 4-hydroxy-3-methoxymandelic acid (VMA, CA metabolites), lipopolysaccharide-phosphate (LPS-P), ALP activity (ALP-A) and Concanavalin A (ConA)-binding rate of ALP (ALP-C) were determined by colorimetric method and lectin (ConA) affinity precipitation method.

**Results:**

More injuries and ONOO^−^, MDA, VMA, LPS-P, ALP-C were increased, ALP-A were decreased in the gut, liver and brain of HCC group, the most in HCC + LDSD group, after treatment with XCHD, all of which were improved. A positive association found between gut-liver-brain injury and ONOO^−^, MDA, VMA, LPS-P, ALP-C, between ONOO^−^, MDA, VMA, LPS-P and ALP-C in the gut, liver and brain, and a negative association found between gut-liver-brain injury and ALP-A, between ALP-A and ONOO^−^, MDA, VMA, LPS-P, ALP-C in the gut, liver and brain.

**Conclusions:**

XCHD can attenuates the vicious circle between the oxidative stress, nitrosative stress, N-glycan deficiency and inactivation of ALP through LPS-CA interactions in gut, liver and brain.

## Background

The applications of XCHD in treatment of tumors have attracted more and more attention. XCHD helped to prevent the development of HCC in patients with cirrhosis, particularly in patients without HBs antigen [1], Shiota *et al.* found that XCHD (TJ-9) reduced the number of preneoplastic cells, inhibited the development of liver tumors, and significantly decreased the formation of 8-hydroxy-2′-deoxyguanosine (8-OHdG), they concluded that XCHD prevents hepatocarcinogenesis in association with inhibition of 8-OHdG formation [2], but few literature covers this field of the efficacy of the XCHD by affecting multiple organ systems.

There is a strong relationship between liver injury and gut injury by portal system and endogenous gut-derived bacterial lipopolysaccharides (LPS), which induces production of catecholamines (CA) in the gut-liver-brain, which are involved in the neuroendocrine and immune effects of LPS, and also contributes to the sensitizing effect of ethanol preexposure on LPS-induced gut-liver-brain damage and ultimately, to the LPS-CA interactions promote hepatocellular carcinoma development [3-9], its mechanism is still unknown.

Main symptoms for the pattern of LDSD are mental depression, sentiment, fatigue, reddish or pale tongue, reduced food intake, fine or fine stringlike pulse.

LDSD has already become common traditional Chinese medicine (TCM) syndrome pattern in clinical, especially in chronic hepatopathy such as chronic hepatitis, cirrhosis and hepatocarcinoma. Chronic stress or depression (squeezing tails, solitary breeding and intermittent fasting) is often used to induce LDSD.

We examined whether XCHD attenuates the vicious circle between the oxidative stress and the ALP inactivation through LPS-CA interactions in gut, liver and brain during CCI_4_ + ethanol-induced mouse HCC with LDSD.

## Methods

### Ethics

This study was approved by Guangdong Science and Technology Committee and Guangdong Management Committee for Medical Experimental Animals.

### General procedure

Chinese Kun Ming (KM) mouse were obtained from the Laboratory Animal Research Center of Guangzhou University of Traditional Chinese Medicine. The animals were housed in a plastic cage in an air-conditioned room at 20 ± 2°C with a humidity 58% ± 5% in a 12-h light and dark cycle with free access to standard rat food and tap water.

### Animals model

A total of 80 male KM mice (cleaning grade, 30 days of age, 18-22 g) were used. The KM mouse were divided into four groups, normal group (10 mice), Hepatocellular carcinoma group (HCC,30 mice), were induced as previously described [10] with some minor modifications by subcutaneously injected with 25% CCI_4_ olive oil solution (5 mL/kg twice per week) and allowed free access to a 8% ethanol solution as drinking fluid for 4 weeks, and allowed free access to a 0.5% CCI_4_-8% ethanol solution as drinking fluid for 20 weeks; HCC + LDSD group (HCC + LDSD, 30 mice), on the basis of HCC model, the mouse were stimulated with the factor of Liver Depression with Spleen Deficiency (LDSD): squeezing tails (30 min/day) for 4 weeks, solitary breeding and intermittent fasting for 20 weeks; HCC + LDSD + XCHD group (XCHD, 30 mice), XCHD was administered by gavage for 8 weeks after 4 weeks of the HCC + LDSD. We recommend 30 mice/model group, this relatively large number of mice is recommended to ensure that enough mice for good statistical analysis, tumor models process often leads to the incidental death of mice.

### Preperation and administration of XCHD

The XCHD consisting of seven herbal medicines (Table 1) and each crude drug was purchased from Hexiang Pharmaceutical Co., Ltd., China. The origin of materials was confirmed and standardized taxonomically by the Traditional Chinese Medicine Herbal Pharmacy, Guangzhou University of TCM. A decoction of XCHD was prepared in our laboratory (Table 1) from a mixture of chopped crude herbs, extracted in distilled water at 100°C, this mixture was then concentrated to a final concentration of 100 crude drug gram per 100 milliliter, was administered by gavage, 8 g/kg per day for 8 weeks, was dried out (3.34 g) in hot (100°C) water bath for quantitative assay of saikosaponin a of the major constituents in XCHD, the procedure is as follows.

**Table 1:** The composition of XCHD

**Latin name**	**Amount (g)**	**Company of purchased**	**Source**
Chai Hu (Radix Bupleuri)	20	Hexiang Pharmaceutical Co., Ltd	China
Huang Qin (Radix Scutellariae baicalensis)	10	Hexiang Pharmaceutical Co., Ltd	China
Dangshen (Codonopsis pilosula)	10	Hexiang Pharmaceutical Co., Ltd	China
Ban Xia (Rhizoma Pinelliae Tematae)	10	Hexiang Pharmaceutical Co., Ltd	China
Gan Cao (Radix Glycyrhizae Uralensis)	5	Hexiang Pharmaceutical Co., Ltd	China
Gan jian (Rhizoma Zingiberis)	5	Hexiang Pharmaceutical Co., Ltd	China
Da Zao (Fruc-tus Zizyphi Jujubae)	6	Hexiang Pharmaceutical Co., Ltd	China
Total	66		

### Quantitative assay of saikosaponin a of the major constituents in XCHD [11,12]

#### Linearity and limits of detection of Saikosaponin a

Standard stock solutions of Saikosaponin a were dissolved in 70% methanol containing 0.1 N sulfate acid and stored below 4°C. Working standard solutions were prepared by serial dilution of stock solutions with 70% methanol containing 0.1 N sulfate acid, heated at 100°C for 3 h, Saikosaponin a were converted completely into saikosaponin b by mild acid treatment, the absorbance of Saikosaponin b was detected by UV-spectrophotometer at 252 nm. Calibration curves of Saikosaponin a were calculated by the absorbance of standard solutions in the concentration of 0–200.00 μg /mL.

### Quantitative assay of saikosaponin a in XCHD

Freeze-dried XCHD were prepared with 70% methanol containing 0.1 N sulfate acid, heated at 100°C for 3 h, Saikosaponin a were converted completely into saikosaponin b by mild acid treatment, the absorbance of Saikosaponin b was detected by UV-spectrophotometer at 252 nm. The amount of Saikosaponin a in XCHD was quantified using the calibration curves of the above-mentioned Saikosaponin a.

### Stability of saikosaponin a in XCHD

The stabilities of Saikosaponin a was examined for 6 days (0, 1, 2, 3, 4 and 5 day respectively) by three sample solution.

### Anatomic analysis

Every mouse were autopsied as quickly as possible after natural death and killed, and photographed, and liver were embedded in paraffin and stained with hematoxylin and eosin. The extent of macroscopically apparent inflammation ulceration and tissue injury was done unblinded by two examiners using a scoring system. This tissue was weighed, processed and the resulting supernatant stored at −20°C for the subsequent determination of ONOO^−^, MDA, VMA, ALP-A, for the assay of ALP-C or for the assay of LPS-P.

### Macroscopic score of gut damage

Intestine macroscopic damage score was calculated as previously described [13] with some minor modifications and graded as 0 = no apparent damage, 1 = slight thickening of intestinal wall, redness, 2 = thickening and adhesions, no apparent ulcers, 3 = adhesions and one or more pin-point ulcers, 4 = adhesions and pin-point ulcers with one or more linear ulcer < 1 cm in length, 5 = one or more linear ulcer > 1 cm in length.

### Macroscopic score of fecal loading in the cecum

Fecal loading in the cecum was graded as 0 = no fecal loading in the cecum, 1 = mild fecal loading in the cecum, 2 = moderate fecal loading in the cecum, 3 = marked fecal loading in the cecum.

### Macroscopic score of liver damage

Iiver macroscopic damage score was graded as 0 = reddish brown in color, soft in consistency, smooth surface, 1 = the liver were enlarged, friable, soft in consistency, 2 = hepatic hyperemia, 3 = an enlarged liver with white focal areas of necrosis, 4 = an enlarged liver with white multifocal areas of necrosis, 5 = hepatic steatosis, the liver were soft, yellow, greasy, enlarged, 6 = the liver is mottled red with bile stained areas, of normal or increased size, 7 = contains visible nodules and fibrosis, 8 = micronodular, yellow, fatty, enlarged, 9 = macronodular, brown, non-greasy, shrunken, cirrhosis, 10 = explanted liver showing small single granules bodies, 11 = explanted liver showing large single granules bodies, 12 = explanted liver showing many small granules bodies, 13 = explanted liver showing many large granules bodies.

### ALP-A and ALP-C in the tissue

Extracts from tissue were prepared by homogenization in 9 g/L NaCl at 4°C using a DY89-1 tissue homogenizer. Tissue homogenate was centrifuged at 10000 r/min for 30 min. Clear supernatant were used to determine the ALP-A and ALP-C by lectin (ConA) affinity precipitation method, the precipitation procedure was performed as previously described [14,15]. The clear supernatant were mixed with 400 μL of an aqueous solution of Con A-Sepharose 6B (5 g/L in distilled water). The mixture was incubated for 30 min at 37°C and centrifuged at 2000 r/min for 15 min. Without disturbing the precipitate, we removed the supernate and measured its ALP-A. ALP-C was calculated by subtracting the corrected value from the total ALP-A, ALP-A was measured with β-glycerophosphate as substrate.

### The ONOO^−^ levels in the tissue

The tissue were initially deproteinized with Somogyi reagent [16] (using 2.0 ml 55 mmol/L NaOH followed by 2.0 ml 75 mmol/L ZnSO_4_), after centrifugation, aliquots of supernatant ONOO^−^ concentration was measured from its absorption at 302 nm by using ϵ_302_ = 1670 mol•L^-1^•cm^-1^[17]. Results were expressed as nmol/L per gram wet tissue.

### The MDA levels in the tissue

Samples were weighed and immediately frozen and stored at −20°C until analysis within 2 wk. Samples were subsequently homogenized in buffer and assayed for MDA content using the thiobarbituric acid (TBA) reaction, as described by Uchiyama and Mihara [18]. with some minor modifications. Briefly, 0.5 mL of homogenate (10% concentration) was mixed with 3 mL of 0.05 N HCL. After addition of 1 mL of 0.67% TBA reagent, the tubes were heated in boiling water for 45 min. The color was formed with 4 mL of n-butanol and centrifuged. The color intensity of the butanol layer was estimated by spectrophotometric absorbance at 535 nm. Results were expressed as pmol/L per gram wet tissue.

### LPS-P levels in the tissue

The protocol was performed as previously described [19,20] with the following modifications. Lipopolysaccharide was extracted by the hot phenol/water procedure. The polysaccharide moiety was separated from the lipid A by hydrolysis of lipopolysaccharide in 10% acetic acid at 100°C for 2.5-4 h. After in separation of lipid A by centrifugation at 1300 x g for 15 min, the lipid A was washed twice with warm water (70-80°C) and once with acetone. Degraded polysaccharide, obtained from the supernatant fluid, was purified by centrifugation (10500 × g, 3 h) and the supernatant was lyophilized, the phosphate content of LPS was determined with phosphomolybdate-blue spectrophotometry. The amount of inorganic phosphate was quantified using the phosphate standard curve, which was obtained by using serial dilutions of 1.00 mM KH_2_PO_4_ standard solution.

### VMA levels in the tissue

The protocol was performed as previously described [21] with the following modifications. VMA is extracted by ethyl acetate, is oxidised with periodic acid to vanillin, and the coloured hydrazone formed from the oxidation product and added 2,4-dinitrophenylhydrazine is measured in alkali at 460 nm. The amount of VMA was quantified using the vanillin standard curve, which was obtained by using serial dilutions of 250 ug/ml vanillin standard solution.

### Statistical analysis

Quantitative differences in ONOO−, MDA, VMA, LPS-P, ALP-A, ALP-C between Normal, HCC, HCC + LDSD and XCHD were evaluated with the unpaired Student’s *T*-test. We evaluated quantitative correlations between the variables with the spearman rank correlation test. P values < 0.05 were considered statistically significant. All analyses were performed using SPSS 17.

## Results

### Linearity and range of detection of saikosaponin a

The linearity of the absorbance at 252 nm (x) versus concentration (y, μg/mL) curve for Saikosaponin a was used to calculate the contents of the main components Saikosaponin a in XCHD. The correlation coefficients(R) of the calibration curves were 0.9985. The regression equations were Y = 369.82 × -2.071 for Saikosaponin a. These results showed that the calibration curve was a good linearity.

### Stability of saikosaponin a in XCHD

The stability test of Saikosaponin a was evaluated using the sample solution for 6 days. In Table 2, sample solution retained a content of 76.92-100.88% as compared with the initial content at 0 day. The RSD values of contents about saikosaponin a compounds in sample solution were with in 15.52%.

**Table 2:** Stability of saikosaponin a compounds for 6 days XCHD (n=3)

**Compounds**	**0 day**		**1 day**		**2 day**		**3 day**		**4 day**		**5 day**	
	**mean±SD (mg/g)**	**RSD (%)**	**mean±SD (mg/g)**	**RSD (%)**	**mean±SD (mg/g)**	**RSD (%)**	**mean±SD (mg/g)**	**RSD (%)**	**mean±SD (mg/g)**	**RSD (%)**	**mean±SD (mg/g)**	**RSD (%)**
Saikosaponin	0.14±0.03	2.19	0.14±0.02	1.38	0.14±0.01	7.85	0.12±0.08	6.77	0.12±0.09	7.99	0.11±0.16	15.52

### Histological and anatomic analysis

**Figure 1:**
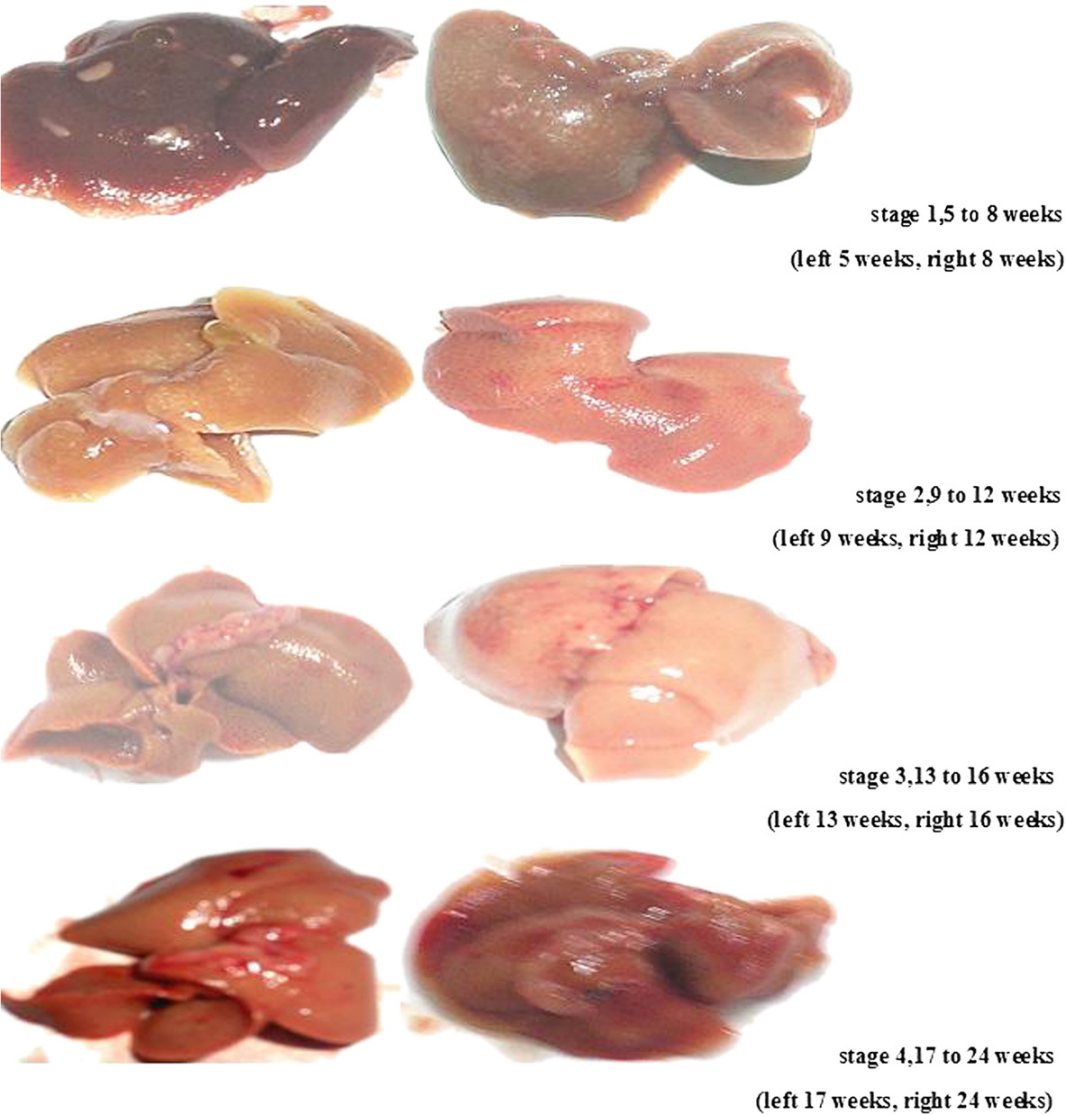
**CCI**_**4**_ **+ ethanol–induced hepatocellular carcinoma (HCC) Chinese Kun Ming (KM) mouse were treated with CCI**_**4**_ **+ ethanol and autopsied after natural death and killed.** Histological examination of liver sections by H&E staining showed that the induction of hepatic cancer involves four stages: stage 1, hepatic steatosis occurred at 5 to 8 weeks; stage 2: hepatic cirrhosis occurred at 9 to 12 weeks; stage 3, hepatic precancerous lesion occurred at 13 to 16 weeks; stage 4, hepatic carcinoma occurred at 17 to 24 weeks. Photomacrographs are liver representative of at least two observations.

During chemical induction of hepatic cancer by CCI_4_ + ethanol, Histological examination of liver sections by H&E staining showed that the induction of hepatic cancer involves four stages: stage 1, hepatic steatosis occurred at 5 to 8 weeks during hepatic carcinoma induction; stage 2, hepatic cirrhosis occurred at 9 to 12 weeks during hepatic carcinoma induction; stage 3, precancerous Lesion of Liver occurred at 13 to 16 weeks during hepatic carcinoma induction; stage 4, hepatic carcinoma occurred at 17 to 24 weeks during hepatic carcinoma induction(Figure 1).

HCC group were significantly more deep red color of liver, the surface of the liver is granular and nodular, a more disordered structure of hepatic lobule, the hepatic cell nuclear anachromasis with nuclear atypia in the H&E stained section. HCC + LDSD group were significantly more dark red color of liver, the surface of the liver is granular and nodular, a more disordered structure of hepatic lobule, the hepatic cell nuclear anachromasis with nuclear atypia in the H&E stained section.

Significantly more injuries in small intestine, cecum, liver(mainly hyperplasia) of HCC, only a slight non-significant reduction was observed in brain weights, compared with normal group, the most in HCC + LDSD group, compared with HCC group, after treatment with XCHD, all of which were improved, compared with HCC + LDSD group. The other organs do not show obvious macroscopic damage (Table 3).

**Table 3:** Effect of XCHD on macrospic gut-liver injury and brain weights

**Groups**	**Dose (gkg**^**-1**^**)**	**n**	**Intestine injury score**	**Cecum injury score**	**Fecal loading in the cecum (score)**	**Colore injury score**	**Liver injury score**	**Brain weights (g)**
Normal	-	6	0.5±0.55	0.67±0.52	0.67±0.52	0.33±0.52	0.5±0.55	0.44±0.04
HCC	-	13	1.77±0.73^b^	1.62±0.51^b^	1.15±0.69	0.46±0.52	9.15±2.08^b^	0.43±0.04
HCC+LDSD	-	9	2.33±0.50^c^	2.33±0.71^c^	1.44±0.73	0.56±0.53	10.67±1.80^c^	0.39±0.06
XCHD	8	14	1.57±0.52^f^	1.50±0.52^f^	1.33±0.52	0.43±0.51	8.21±2.08^f^	0.43±0.05

^b^<0.01 Compare with normal; ^c^P <0.05 Compare with HCC; ^f^<0.01 Compare with HCC+ LDSD.

### Effects of XCHD on MDA, ONOO^−^, VMA, LPS-P, ALP-A, ALP-C in the gut, liver and brain

ONOO^−^, MDA, VMA, LPS-P, ALP-C were increased, ALP-A were decreased in the gut, liver and brain of HCC group, compared with normal group, the most in HCC + LDSD group, compared with HCC group, after treatment with XCHD, all of which were improved, compared with HCC + LDSD group (Table 4).

**Table 4:** **Effects of XCHD on MDA, ONOO**^**-**^**, VMA, LPS-P, ALP-A and ALP-C in the gut, liver and brain**

**Groups**	**Dose (g.kg**^**-1**^**)**	**Gut (n)**	**Liver (n)**	**Brain (n)**
**MDA (nmol/L/g)**				
Normal	-	5.48±1.78(6)	300.86±29.16(6)	68.34±13.22(6)
HCC	-	62.75±19.80(9)^b^	318.21±23.27(6)	126.39±30.92(8)^b^
HCC+LDSD	-	171.67±42.24^d^	360.87±48.02(6)^c^	180.72±27.05(8)^d^
XCHD	8	58.69±17.99^f^	319.75±19.28(7)	127.06±24.33(8)
**ONOO**^**-**^** (mmol/L/g)**				
Normal	-	7.39±0.66(6)	12.87±0.95(6)	3.99±24.33(8)^f^
HCC	-	10.61±0.85(13)^b^	16.40±0.80(13)^b^	7.46±1.42(13)^b^
HCC+LDSD	-	15.19±1.78(9)	19.86±0.80(9)	12.90±0.48(9)^d^
XCHD	8	10.31±1.12(14)^f^	14.15±0.92(9)	12.90±0.48(9)
**VMA (ug/ml/g)**				
Normal	-	6.78±1.09(6)	32.46±5.64(6)	1.76±0.96(6)
HCC	-	9.94±0.10^b^	51.01±5.06(9)^b^	3.99±0.46(6)^b^
HCC+LDSD	-	19.79±0.28(6)^d^	86.66±5.06(9)d	11.81±6.60(6)^c^
XCHD	8	10.52±0.56(6)^b^	74.38±10.20(14)^f^	2.92±2.82(6)^f^
**LPS-P (mmol/L/g)**				
Normal	-	0.55±0.04(6)	0.36±0.11(6)	0.052±0.003(6)
HCC	-	0.66±0.06(6)^b^	0.86±0.09(13)^b^	0.060±0.004(6)^b^
HCC+LDSD	-	0.76±0.06(6)^d^	1.30±0.43(6)^d^	0.075±0.003(6)^d^
XCHD	8	0.57±0.05(6)^f^	0.71±0.12(14)^f^	0.059±0.009(6)^f^
**ALP-A (μ)**				
Normal	-	131.98±30.86(6)	140.17±8.74(6)	29.60±2.41(6)
HCC	-	55.52±8.87(6)^b^	53.70±7.78(6)^b^	27.75±4.66(6)^b^
HCC+LDSD	-	35.94±8.77(6)^d^	38.73±7.78(6)^d^	20.27±±4.65(6)^d^
XCHD	8	94.16±12.35(6)^f^	64.13±7.52(6)^f^	39.37±6.12(6)^f^
**ALP-C (%)**				
Normal	-	8.69±3.75(6)	10.01±1.24(6)	26.06.70(6)
HCC	-	47.32±14.75(6)^b^	56.89±9.98(6)^b^	37.84±6.95(6)^b^
HCC+LDSD	-	78.47±18.41(6)^d^	75.37±14.87(6)^c^	80.0±5.28(6)^d^
XCHD	8	20.73±4.82(6)f	32.73±4.49(6)^f^	45.73±11.43(6)

^a^P <0.05, ^b^P<0.01 Compare with normal; ^c^P <0.05, ^d^P <0.01 Compare with HCC; ^e^P <0.05, ^f^P <0.01 Compare with HCC+LDSA.

### The correlation coefficient between gut injuries, liver injuries, brain injuries, MDA, ONOO^−^, VMA, LPS-P, ALP, ALP-C

Correlative analysis showed that there was a positive association between gut-liver-brain injury and ONOO^−^, MDA, VMA, LPS-P, ALP-C, between ONOO^−^, MDA, VMA, LPS-P and ALP-C in the gut, liver and brain, there was a negative association between gut-liver-brain injury and ALP-A, between ALP-A and ONOO^−^, MDA, VMA, LPS-P, ALP-C in the gut, liver and brain (Tables 5, 6, 7 and 8).

**Table 5:** The correlation coefficient between gut injury, liver injury, brain injury, MDA, ONOO, CMA, LPS-P, ALP-A and ALP-C in the gut

	**n**	**ALP-A**	**ALP-C**	**MDA**	**ONOO**	**LPS-P**	**VMA**
ALP-A	24		−0.68^b^	−0.79^b^	−0.78^b^	−0.71^b^	−0.71^b^
ALP-C	24			0.49^b^	0.57^b^	0.63^b^	0.59^b^
MDA	24				0.94^b^	0.73^b^	0.92^b^
ONOO^-^	24					0.70^b^	0.91^b^
LPS-P	24						0.79^b^
Intestine	24	−0.87^b^	0.56^b^	0.78^b^	0.80^b^	0.64^b^	0.69^b^
Cecum injury	24	−0.60^b^	0.66^b^	0.53^b^	0.63^b^	0.63^b^	0.66^b^
Fecal loading in the cecum	24	−0.60^b^	0.66^b^	0.53^b^	0.63^b^	0.63^b^	0.26
Colorectal injury	24	0.03	0.20	0.01	0.02	0.25	0.17
Liver injury	24	−0.80^b^	0.68^b^	0.68^b^	0.73^b^	0.54^b^	0.63^b^
Brain weights	24	0.46^a^	−0.42^a^	−0.52^b^	−0.41^a^	−0.53^b^	0.55^b^

^a^P<0.05, ^b^P<0.01, test of significance of correlation coefficient.

**Table 6:** **The correlation coefficient between gut injury, liver injury, brain injury, MDA, ONOO**^**-**^**, VMA, LPS-P, ALP-A and ALP-C in the liver**

	**n**	**ALP-A**	**ALP-C**	**MDA**	**ONOO**	**LPS-P**	**VMA**
ALP-A	24		−0.86^b^	−0.42^a^	−0.68^b^	−0.75^b^	−0.83^b^
ALP-C	24			0.40^a^	0.60^b^	0.74^b^	0.65^b^
MDA	24				0.51^b^	0.44^a^	0.60^b^
ONOO^-^	24					0.63^b^	0.80^b^
LPS-P	24						0.71^b^
Intestine	24	−0.80^b^	0.80^b^	0.50^b^	0.70^b^	0.63^b^	0.73^b^
Cecum injury	24	−0.65^b^	0.51^b^	0.30	0.60^b^	0.34	0.76
Fecal loading in the cecum	24	−0.33	0.32	0.24	0.17	0.40^a^	0.36^a^
Colorectal injury	24	−0.09	−0.32	0.02	0.18	−0.11	0.17
Liver injury	24	−0.94^b^	0.87^b^	0.43^a^	0.55^b^	0.73^b^	0.75^b^
Brain weights	24	0.53^b^	−0.39^a^	−0.49^b^	−0.51^b^	−0.22	−0.60^b^

^a^P<0.05, ^b^P<0.01, test of significance of correlation coefficient.

**Table 7:** **The correlation coefficient gut injury, liver injury, brain injury, MDA, ONOO**^**-**^**, VMA, LPS-P, ALP-A and ALP-C in the brain**

	**n**	**ALP-A**	**ALP-C**	**MDA**	**ONOO**	**LPS-P**	**VMA**
ALP-A	24		0.30	−0.25	−0.49^b^	−0.42^a^	−0.52^b^
ALP-C	24			0.57	0.51^b^	0.51^b^	0.25
MDA	24				0.76^b^	0.71^b^	0.46^a^
ONOO^-^	24					0.84^b^	0.66^b^
LPS-P	24						0.63^b^
Intestine	24	−0.26	0.39^a^	0.73^b^	0.80^b^	0.61^b^	0.39
Cecum injury	24	−0.36	0.38^a^	0.50^b^	0.69^b^	0.71^b^	0.66^b^
Fecal loading in the Cecum	24	−0.09	0.44^a^	−0.05	0.12	0.23	−0.01
Colorectal injury	24	−0.09	0.20	−0.05	0.12	0.23	−0.01
Liver injury	24	−0.17	0.53^b^	0.76^b^	0.65^b^	0.55^b^	0.50^b^
Brain weights	24	0.05	−0.63^b^	−0.39^a^	−0.50^b^	−0.50^b^	−0.19

^a^P<0.05, ^b^P<0.01, test of significance of correlation coeficient.

**Table 8:** **The correlation coefficient between MDA, ONOO**^**-**^**, VMA, LPS-P, and ALP-C in the gut, liver and brain**

	**n**	**Liver MDA**	**Liver ONOO**	**Liver VMA**	**Liver LSP-P**	**Liver ALP-A**	**Liver ALP-C**
Liver							
	24		0.51^b^	0.60^b^	0.44^a^	0.42^a^	0.40^a^
	24			0.80^b^	0.63^b^	−0.63^b^	0.60^b^
	24				0.71^b^	−0.83^b^	0.65^b^
	24					−0.75^b^	0.74^b^
	24						−0.86^b^
	24						
Gut							
	24	0.62^b^	0.84^b^	0.86^b^	0.84^b^	−0.73^b^	0.69^b^
	24	0.55^b^	0.81^b^	0.90^b^	0.84^b^	−0.80^b^	0.68^b^
	24	0.60^b^	0.86^b^	0.93^b^	0.76^b^	−0.73^b^	0.60^b^
	24	0.41^a^	0.74^b^	0.69^b^	0.59^b^	−0.64^b^	0.67^b^
	24	−0.56^b^	−0.72^b^	−0.76^b^	−0.71^b^	0.82^b^	−0.89^b^
	24	0.29	0.63^b^	0.64^b^	0.56^b^	−0.76^b^	0.66^b^
Brain							
	24	0.55^b^	0.66^b^	0.80^b^	0.74^b^	−0.83^b^	0.69^b^
	24	0.61^b^	0.84^b^	0.95^b^	0.73^b^	−0.74^b^	0.62^b^
	24	0.20	0.60^b^	0.67^b^	0.54^b^	−0.53^b^	0.43
	24	0.52^b^	0.89^b^	0.85^b^	0.64^b^	−0.71^b^	0.58^b^
	24	−0.29	−0.47^a^	−0.34	−0.53^b^	0.21	−0.41^a^
	24	0.35^a^	0.36^a^	0.68^b^	0.35^a^	−0.62^b^	0.28

^a^P<0.05, ^b^P<0.01, test of significance of correlation coeficient.

## Discussion

In our present study, more injuries and ONOO^−^, MDA, VMA, LPS-P, ALP-C were increased, ALP-A were decreased in the gut, liver and brain of HCC group, compared with normal group, the most in HCC with Liver Depression and Spleen Deficiency(LDSD) group, compared with HCC group, after treatment with XCHD, all of which were improved, compared with HCC + LDSD group (Tables 3 and 4). A positive association found between gut-liver-brain injury and ONOO^−^, MDA, VMA, LPS-P, ALP-C, between ONOO^−^, MDA, VMA, LPS-P, ALP-C, and a negative association found between gut-liver-brain injury and ALP-A, between ALP-A and ONOO^−^, MDA, VMA, LPS-P, ALP-C in the gut, liver and brain (Tables 5, 6, 7 and 8).

Yu LX *et al.*[7] found endotoxin accumulation prevents carcinogen-induced apoptosis and promotes liver tumorigenesis. Zhou L *et al.*[22] and Tang CH *et al.*[23] found oxidative stress-induced 1, N6-ethenodeoxyadenosine adduct formation and uncontrolled nitrosative stress contributes to hepatocarcinogenesis. Xiang Q *et al.*[24] found LPS-induced hepatotoxicity possibly by cytotoxic effects of oxygen free radicals, NO and cytokines, carnosic acid successfully and dose dependently attenuates the effects of LPS.von Montfort C *et al.*[5] found the sympathetic hormone epinephrine preexposure enhances LPS-induced liver damage. Schäper J *et al.*[25] found Regional sympathetic blockade significantly attenuated the endotoxin-induced increases in gut epithelial permeability, expression of nitric oxide synthase, macrophage infiltration, and lipid peroxidation. Adham KG *et al.*[26] found lipopolysaccharide stimulated the biosynthesis of norepinephrine and dopamine in all brain regions, was completely abolished by pretreatment with indomethacin.

The toxic moiety of Lipopolysaccharide (LPS) is the well-preserved lipid A part. Lipid A contains two phosphate groups attached to diglucosamine, which are crucial for the many biological activities of LPS [27]. Radiodetoxified lipopolysaccharide fails to activate the hypophyseal-pituitary-adrenal axis [28] and alkaline Phosphatase (ALP) was able to dephosphorylate LPS and prevents inflammation [29].

ALP are important defense factor in protecting the gut-liver-brain multiple organ against bacterial infection [30-32]. Halling Linder C *et al.*[33] found significantly different catalytic properties among the bone ALP isoforms due to structural differences in N-glycosylation. The changes in protein N-glycosylation play an important role in the pathogenesis and progression of various liver diseases [34]. Cottalasso D *et al.*[35] found ethanol administration induces a marked decrease of dolichyl phosphate (a glycosyl sugar carrier for N-glycosylation of proteins) in rat liver microsomes and Golgi apparatus. Yasuda J *et al.*[36] found ROS result in the cleavage of oligosaccharides of glycoproteins including transferrin may contribute the development of acute hepatitis.

We had previously found [14] that ConA, PSA, PNA, and LCA binding rate of serum amylase were significantly higher in HCC and hepatocirrhosis patients than hepatitis patients and normal controls. PSA and LCA binding rate of serum amylase were significantly higher in HCC + LDSD patients than those with liver and kidney Yin deficiency. HCC + LDSD patients had higher ConA binding rate of serum amylase compared with those with QI and blood stasis. A positive correlation was found between PSA, LCA, and PNA binding rate of serum amylase and MDA.

## Conclusions

Above all these observations suggest that there was a significant increase in gut-liver-brain injury of CCI_4_ + ethanol-induced mouse HCC, it is associated with the vicious circle between the oxidative stress, nitrosative stress and N-glycan deficiency and inactivation of ALP in gut, liver and brain through LPS-CA interactions.

LDSD significantly increased gut-liver-brain injury in CCI_4_ + ethanol-induced mouse HCC, it's maily because LDSD can significantly aggravate the vicious circle between the oxidative stress, nitrosative stress and N-glycan deficiency and inactivation of ALP in gut, liver and brain through LPS-CA interactions.

XCHD significantly decreased gut-liver-brain injury in CCI_4_ + ethanol-induced mouse HCC, it's maily because XCHD can significantly attenuate the vicious circle between the oxidative stress, nitrosative stress and N- glycan deficiency and inactivation of ALP in gut, liver and brain through LPS-CA interactions, by increased N-glycan and activation of ALP, decreased LPS toxicity, oxidative stress, nitrosative stress, CA (VMA), ultimately to improved the gut, liver and brain injury and the dysfunction of gut-liver-brain interactions.

## Abbreviations

XCHD: Xiaochaihu decoction, which is a classic and famous prescription founded by Zhang Zhong-jing, which is a main prescription treating Shaoyang Syndrome in Shanghanlun; LDSD: Liver depression and spleen deficiency, Main symptoms for the pattern of LDSD are mental depression, sentiment, fatigue, reddish or pale tongue, reduced food intake, fine or fine stringlike pulse; ALP: Alkaline phosphatase; ALP-A: Alkaline phosphatase activity; ALP-C: Concanavalin A (ConA)-binding ratios of alkaline Phosphatase; LPS: Lipopolysaccharide; LPS-P: Lipopolysaccharide-phosphate; VMA: 4-hydroxy-3-methoxymandelic acid, the end product of both epinephrine and norepinephrine catabolism, was used as an index of catecholamine activity; ONOO−: Peroxynitrite, is a reactive oxygen species that reacts quickly with nitric oxide; MDA: Malondialdehyde, is a reactive oxygen species, could reflect oxidative damage to lipids.

## Competing interests

We declare that we have no competing interests.

## Authors’ contributions

X-QL conceived of the study, carried out the molecular studies, performed the statistical analysis and drafted the manuscript. X-JH carried out the animal experiments, participated in the molecular studies and helped to draft the manuscript, H-XX and X-YZ carried out to evaluate the chemical constituents of Xiaochaihu Decoction (XCHD). All authors read and approved the final manuscript.

## Authors’ information

Xiaoqiu Liu, Xiaojian Hu, Hong-Xing Xu, Xiao-Ying Zeng. The vicious circle between the oxidative stress and the ALP inactivation through LPS-catecholamines interactions.

## Pre-publication history

The pre-publication history for this paper can be accessed here:

http://www.biomedcentral.com/1472-6882/13/375/prepub
